# The effect of using virtual reality technology on anxiety and vital signs before surgery in patients undergoing open heart surgery

**DOI:** 10.1186/s13741-023-00354-8

**Published:** 2023-11-24

**Authors:** Ameneh Amiri, Rostam Jalali, Nader Salari

**Affiliations:** Kermanshah University of Medical Sciences, Kermanshah, Iran

**Keywords:** Virtual reality, Anxiety, Open heart surgery, Immersion

## Abstract

**Introduction:**

Preoperative anxiety is one of the most common psychological problems in open-heart surgery patients. Not controlling this problem can negatively the operation outcome and the patient’s physical condition. Among various training methods and tools introduced to deal with this issue, the ideal method still remains unknown. Therefore, the present study was to determine the effect of using virtual reality technology on preoperative anxiety in patients undergoing open heart surgery.

**Materials and methods:**

The participants of this interventional-educational study included 60 patients who were candidates for open heart surgery. The samples were randomly divided into two groups virtual reality(*n* = 30)and ordinary video (*n* = 30). For the virtual reality group, a virtual reality film and for the ordinary video group, an ordinary video of the physical space and operating room staff were displayed the day before the operation. Patients’ anxiety in both groups was assessed using the Spielberger State-Trait Anxiety Inventory (STAI) before and after the intervention. Data analysis was performed using the SPSS software version 25.

**Results:**

The mean anxiety score before the intervention was 55.8 and 58.33 in the virtual reality group and the ordinary video group, respectively. After the intervention, it reached 38.60 in the virtual reality group and 45.13 in the control group. There was no statistically significant difference between the anxiety scores of the subjects in the virtual reality and ordinary video groups before the intervention (*p* > 0.05). However, the difference between the anxiety scores of the subjects in the virtual reality and ordinary video groups after the intervention was significant (*p* < 0.05).

**Conclusion:**

Although virtual reality and ordinary video interventions effectively reduce anxiety in heart surgery patients, virtual reality seems to lower anxiety in heart surgery patients by diverting attention from external stimuli and immersing the person in the virtual world more than ordinary video.

## Introduction

Coronary artery disease (CAD) is one of the leading causes of morbidity and mortality in both developing and developed countries (Ramesh et al. 2017). Surgery is the only treatment of choice in many cardiovascular patients (Quds et al. 2019). Research has shown that more than 515,000 coronary artery bypass surgeries are performed annually in the United States and 17,000 in Australia (Malmir et al. 2015). This surgery is associated with several psychological complications for patients. Among the mental disorders before and after surgery, depression and anxiety are more common and important (Mousavi et al. 2013). Anxiety is an emotional state of fear, nervousness, and worry about threatening events associated with physiological alertness which is accompanied by restlessness, fatigue, problems in concentration, and muscular tension (Almalki et al. 2017). Waiting for heart surgery, hospitalization, fear of death, knowing a person who has died of the same disease in the past, and generally fear of the unknown cause anxiety in the patient (Khodarahi 2021). Studies conducted in the European region showed that the prevalence of preoperative anxiety among surgical patients varied from 27 to 80% (Prado-Olivares and Chover-Sierra 2019). Studies conducted in India revealed that the prevalence of preoperative anxiety varied from 47% to 70.3% while the prevalence of preoperative anxiety in Pakistan ranged from 62 to 97% (Jafar and Khan 2009). Anxiety can reduce wound healing, increase the infection risk, alter the immune response, and lead to electrolyte and fluid imbalances, and changes in sleep patterns. These factors may prolong hospital stays and delayed discharge (Moghadam et al. 2016). Therefore, a thorough physical and fitness examination of these patients is very important (Mousavi et al. 2013). People who are physically and mentally prepared for surgery usually experience easier surgery due to more relaxation. In addition, relaxation in these patients leads to pain tolerance, reduced need for medication, and shorter hospital stays (Movahed et al. 2016). Today, medication is commonly used to reduce preoperative anxiety. Nevertheless, these drugs have side effects such as drowsiness and suppression of the immune system and sometimes lead to adverse reactions (Khodarahi 2021). Providing non-pharmacological measures can relieve anxiety without serious side effects and is usually less risky for the patient (Quds et al. 2019).

Virtual reality is an interactive form of distraction where a human becomes an active participant in a virtual environment (Wong et al. 2021). In this system, a virtual world is created in a three-dimensional space that stimulates the user’s senses, such as sight and hearing. In this way, the human brain believes that he is in that environment, and feels that he is immersed in that environment (Nazemi et al. 2019). Virtual reality has been used as a new tool in various types of rehabilitation (Schultheis and Rizzo 2011), neuroscience treatments (Bohil et al. 2011), mental disorders such as pain (Mclay 2013), stress (Juan et al. 2014), fears (Carroll et al. 2015), and common anxieties (Maples-Keller et al. 2017; Krijn et al. 2014). In addition, it is highly considered in modern medical education, such as surgery training (Weller 2016). So far, several studies have been performed on patients undergoing open heart surgery using tools such as face-to-face training, Benson relaxation, multimedia, and aromatherapy to reduce anxiety (Malmir et al. 2015; Mousavi et al. 2013; Jafar and Khan 2009; Movahed et al. 2016). However, the ideal way to give information to these patients and reduce their anxiety remains unknown (Jafar and Khan 2009). Therefore, the present study was conducted to determine the effect of using virtual reality technology on preoperative anxiety in patients undergoing open heart surgery.

## Materials and methods

The present study was conducted based on the interventional-educational approach. The statistical population of this study consisted of patients who were candidates for open heart surgery referred to the hospital, in which 60 patients were randomly assigned to two groups of virtual reality and ordinary video. The random allocation was concealed by applying, the method of opaque sealed envelopes with a random sequence. After the visit, the patients received a number according to the priority of their arrival at the center, that corresponded to the numbers on the sealed envelopes. There was one of codes A and B inside the sealed envelopes. This sequence was identified by a non-sampler using www.Randomizer.org. Each of the codes represented the study group in which the individual was placed. Code A represented the virtual reality intervention group and Code B represented the conventional video intervention group. Inclusion criteria were being candidates for open heart surgery, communication skills, literacy, the age range of 30–70 years, lack of severe hearing and vision problems, willingness to participate in the study, and no history of mental illness. Exclusion criteria included being in an emergency situation (e.g., cardiac arrhythmia and respiratory distress) and using and receiving narcotics and sedatives. Data collection tools were (1) demographic characteristics questionnaire, which included age, sex, marital status, level of education, medical history, and smoking according to the research objectives. (2) The Spielberger State-Trait Anxiety Inventory (STAI); this tool consists of 20 items to assess a person’s feelings at “the moment and the time to respond.” The four-choice expressions are scored as very low, low, high, and very high. The minimum and maximum score obtained from the questionnaire is 20–80. Scoring is as follows: mild anxiety rate from 20 to 31, moderate to low anxiety: 32–32, moderate to high anxiety 43–53, moderately severe 54–64, severe: 65–75, and very severe:76 and above. In Mehram’s (Borang et al. 2017) study, the internal consistency of both scales was calculated via Cronbach’s α on a group of 600 people. The Cronbach’s α coefficients of the State Anxiety Scale and the Breggy Anxiety Scale were reported to be 0.91 and 0.92, respectively. For the total test, Cronbach’s α coefficient is 0.94. In this study, The content of both videos is the same, but the ordinary video was recorded with a normal camera and the virtual reality was recorded with a Nikon 360° camera. The duration of the video is 4 minutes and 35 s.

 In the video, the physical space of the operating room was shown. The video starts from the entrance door of the operating room and the camera moves from the corridor and reaches the room where the operation is performed. Next, the researcher entered the room and explained the application of the devices used in open heart surgery, (i.e., the ventilator, cardiopulmonary pump, monitor, and operating table) and how to place the patient on the operating bed to participants in simple language. At the end of the video, the researcher explained that the patient was transferred to the intensive care unit (ICU) after the operation, wherein treatment was continued. The virtual reality video was displaced to the samples, using the TSCO virtual reality glasses (model TVR 568). To this end, the video was placed in the virtual reality glasses and displayed with the phone device along with the virtual reality video player software. For the normal video group, the same video made by the iPad was displayed. To start the study, the researcher referred to the men’s and women’s cardiac surgery wards of the Cardiovascular Hospital the day before the operation. After explaining the study’s objectives and obtaining informed consent to participate in the study, the participants were asked to complete the demographic information form, and the medical record was prepared. Afterward, the SATI was provided to the samples to complete by themselves, and the vital signs of the samples were measured by the researcher and recorded. In the next step, an educational film was displayed for two groups. Some of the samples in both groups requested to watch the videos again, which was done. Finally, on the day of surgery, the researcher reappeared in the wards of the study center. About 2 h before receiving the prescribed drugs to prepare the patient for surgery, the SATI was completed by both groups, and the researcher measured and recorded the vital signs. Finally, the data were entered into SPSS-25 software.

## Results

In this study, the mean age of the samples in the virtual reality and the ordinary video groups was 56.1 (SD = 7.6)(range 30–70) and 56.7 (range 30–70) years (SD = 6.9), respectively. Also, 63.3% of patients were male (*n* = 38), 91.6% married (*n* = 55), and 33.3% were smokers (*n* = 20). About 70% (42 patients) had high blood pressure and 8.3% (5 patients) had diabetes (Table 1). The statistical analysis results (independent T) showed that the mean score of anxiety in the group of virtual reality and ordinary video before the intervention was not statistically significant (*p* value = 0.19). The mean score of overt anxiety in the virtual reality group before the intervention was 55.80, which after the intervention reached 38.60, and in the normal video group from 58.33 to 45.13. The independent *t* test results showed a statistically significant difference between the anxiety scores of the subjects in the virtual reality and ordinary video groups after the intervention (*p* value < 0.05). Also, since the average anxiety score in the virtual reality group is lower, this intervention seems to be more effective than ordinary video (Table 2). The results of comparing the mean of clinical variables (i.e., blood pressure, heart rate, respiration rate) before and after the intervention in the two groups of virtual reality and ordinary video revealed a statistically significant difference between the mean of clinical variables in the two groups before and after the intervention (*p* value < 0.05) (Table 3).

**Table 1:** Demographic information of research samples

Variables		Ordinary video group		Virtual reality group	
		Number	Percent	Number	Percent
Gender	Male	19	63.3	19	63.3
	Female	11	36.7	11	36.7
	Total	30	100	30	100
Education	High school	16	53.3	16	53.3
	Diploma	9	30	9	30
	Bachelor and higher	5	16.7	5	16.7
	Total	30	100	30	100
Marry	Single (single, divorced, widowed)	1	3.3	4	13.3
	Married	29	96.7	26	86.7
	Total	30	100	30	100
Smoking	Yes	7	23.3	13	43.3
	No	23	76.7	17	56.7
	Total	30	100	30	100
Hypertension	Yes	25	83.3	17	56.7
	No	5	16.7	13	43.3
Diabetes	Yes	-	-	5	16.7
	No	30	100	25	83.3

**Table 2:** Comparison of the mean anxiety score of patients before and after the intervention

Variable			*p* value	SD	Mean
Anxiety	Virtual reality	Before	55.80	49.6	0.001
		After	38.60	64.5	
	Ordinary video	Before	58.33	8.28	0.001
		After	45.13	9.15

**Table 3:** Results of comparing the mean of clinical variables before and after the intervention

Variable	Virtual reality		Ordinary video		*p* value
	Before	After	Before	After	
	Mean ± SD	Mean ± SD	Mean ± SD	Mean ± SD	
Heart rate	69.33 ± 5.89	66.30 ± 5.05	72.90 ± 5.95	68.90 ± 5.16	0.001
Systolic blood pressure	124 ± 11.01	122 ± 9.28	131 ± 10.73	128 ± 9.70	0.001
Diastolic blood pressure	93.83 ± 9.59	87.86 ± 7.04	97.86 ± 9.91	93.13 ± 7.44	0.001
Respiratory rate	16 ± 0.98	15.40 ± 0.81	15.20 ± 1.29	14.60 ± 1.16	0.001

## Discussion

The is study aimed to investigate the effect of using virtual reality technology on preoperative anxiety in patients undergoing open heart surgery. The results showed that the mean score of anxiety in the virtual reality and ordinary video groups before the intervention was not statistically significant and indicated high levels. Anxiety in patients was a candidate for preoperative open heart surgery. Studying preoperative anxiety surveys in patients undergoing coronary artery bypass surgery, Olivares (Prado-Olivares and Chover-Sierra 2019) showed that patients undergoing open heart surgery experienced significant preoperative anxiety. This result is consistent with the findings of the present study. Cardiac surgery is a major source of stress for patients, and for clients, the heart determines life and death. Fear of the unknown causes anxiety in patients who are candidates for open heart surgery. In the present study, providing training through virtual reality technology effectively reduced preoperative anxiety, and there was a significant difference between the mean anxiety scores of the subjects before and after the intervention. In another study, Łuczak (Łuczak et al. 2021) studied the effect of virtual reality on pain and anxiety in patients undergoing cystoscopy. After the intervention, the anxiety level in the virtual reality group was significantly reduced. The results of the present study are in line with this study. In a review study, Ioannou (Ioannou et al. 2020) showed the high efficiency of virtual reality in the management of anxiety symptoms, depression, fatigue, and pain, the intervention versus control (e.g., standard care). Virtual reality is the complete immersion of the patient in a virtual world in which users believe that they are actually present in the environment in which they are portrayed. This treatment puts the patient psychologically in a good position and appropriate physiological reactions to deal with stress, depression, and anxiety. One of the main reasons for the positive effectiveness of this technology is the exciting audio-visual nature and simulations. Therefore, knowing that it is virtual, the patient tries to adapt to the environment by being in that environment. Also, he tries to establish constructive interaction, to overcome the limitations existing in the real world, and to open a new horizon for itself. Regarding the effect of normal video on anxiety in patients undergoing open heart surgery, present study results showed a statistical difference between the mean anxiety scores of patients before and after the intervention, This difference indicates the effectiveness of Ordinary video training in reducing preoperative anxiety. Haddad (Haddad et al. 2018) investigated the effectiveness of nurse‐led video interventions on anxiety in patients having percutaneous coronary. The results showed that the mean anxiety score for the intervention group was significantly lower than the comparison group. This finding is in line with the results of the present study. Possible reasons for this result are the appropriate number of samples, the correct sampling method, and the correct content used in the educational video. In another study, Monfared (Monfared and Dehghanzadeh 2021) investigated the effect of film education on the level of anxiety in patients undergoing coronary angiography. The results showed a statistical difference between the mean anxiety score before and after the intervention with the educational film. After the training, the anxiety score had risen from 43.03 to 35.06. Educational and animated videos can help convey a message to the learner by stimulating the auditory and visual senses. It can also help the patient learn more deeply by affecting all three areas of learning (e.g., knowledge, emotional, and motor psychology). Therefore, it is recommended to incorporate this method into pre-surgery education in patients. Since one of the most important roles of nurses is patient education, nurses are recommended to pay special attention to providing film education as a supplement to oral education. The results showed a statistically significant difference between the mean of clinical variables (i.e., blood pressure, heart rate, respiration rate) of the subjects in the virtual reality group and normal video, before and after the intervention, This difference suggests the effectiveness of these two methods in reducing preoperative anxiety. Shahmari (Shahmari et al. 2016) investigated the effect of native language film education on vital signs of anxiety in coronary angiography patients. The analysis results showed that in both intervention and control groups, vital signs improved significantly over time. This result is in line with those of the present study, which indicates that fear and anxiety increase the body’s physiological activities, including vital signs and cardiac output, as, very harmful factors for patients with cardiovascular problems.

Overall, it is concluded that educating patients positively affects vital signs by increasing their awareness and reducing their anxiety.

## Limitations

1. The patients’ reluctance to participate in the study: The researcher, attracted the cooperation of the patients by explaining the study’s objectives.
2. Patients’ inability to participate in the study due to illness: The patients participated in the study as soon as they declared their readiness.

## Conclusion

The study results show the effectiveness of virtual reality and educational video methods in reducing preoperative anxiety. Hence, these non-invasive and non-pharmacological methods can be used to control the anxiety of patients who are candidates for surgery.

## Data Availability

Datasets are available through the corresponding author upon reasonable request.
